# Evaluation of absorption, distribution, metabolism, and excretion of [^14^C]-rucaparib, a poly(ADP-ribose) polymerase inhibitor, in patients with advanced solid tumors

**DOI:** 10.1007/s10637-019-00815-2

**Published:** 2019-06-27

**Authors:** Mingxiang Liao, Simon Watkins, Eileen Nash, Jeff Isaacson, Jeff Etter, Jeri Beltman, Rong Fan, Li Shen, Abdul Mutlib, Vendel Kemeny, Zsuzsanna Pápai, Pascal van Tilburg, Jim J. Xiao

**Affiliations:** 1 Clovis Oncology, Inc., 500 Flatiron Pkwy, Suite 100, Boulder, CO 80301 USA; 2 Frontage Laboratories, Inc., 700 Pennsylvania Dr, Exton, PA 19341 USA; 3 PRA Health Sciences, Rottenbiller utca 13, Budapest, H-1077 Hungary; 4 State Health Center, Róbert Károly krt. 44, Budapest, 1134 Hungary; 5 PRA Health Sciences, Bioanalytical Laboratory NL, Amerikaweg 18, 9407 TK Assen, The Netherlands

**Keywords:** Human ADME study, Metabolite identification, Mass balance, Pharmacokinetics, PARP inhibitor, Rucaparib

## Abstract

Rucaparib, a poly(ADP-ribose) polymerase inhibitor, is licensed for use in recurrent ovarian, fallopian tube, or primary peritoneal cancer. We characterized the absorption, distribution, metabolism, and elimination of rucaparib in 6 patients with advanced solid tumors following a single oral dose of [^14^C]-rucaparib 600 mg (≈140 μCi). Total radioactivity (TRA) in blood, plasma, urine, and feces was measured using liquid scintillation counting. Unchanged rucaparib concentrations in plasma were determined using validated liquid chromatography with tandem mass spectrometry. Maximum concentration (C_max_) of TRA and unchanged rucaparib in plasma was 880 ng Eq/mL and 428 ng/mL, respectively, at approximately 4 h post dose; terminal half-life was >25 h for both TRA and rucaparib. The plasma TRA-time profile was parallel to yet higher than that of rucaparib, suggesting the presence of metabolites in plasma. Mean blood:plasma ratio of radioactivity was 1.0 for C_max_ and 0.8 for area under the concentration-time curve from time zero to infinity. Mean postdose recovery of TRA was 89.3% over 12 days (71.9% in feces; 17.4% in urine). Unchanged rucaparib and M324 (oxidative metabolite) were the major components in plasma, contributing to 64.0% and 18.6% of plasma radioactivity, respectively. Rucaparib and M324 were the major rucaparib-related components (each ≈7.6% of dose) in urine, whereas rucaparib was the predominant component (63.9% of dose) in feces. The high fecal recovery of unchanged rucaparib could be attributed to hepatic excretion and/or incomplete oral absorption. Overall, these data suggest that rucaparib is eliminated through multiple pathways, including metabolism and renal and biliary excretion.

**Electronic supplementary material:**

The online version of this article (10.1007/s10637-019-00815-2) contains supplementary material, which is available to authorized users.

## Introduction

Rucaparib is a potent, oral, small molecule inhibitor of poly(ADP-ribose) polymerase (PARP) 1, PARP2, and PARP3 [1]. In tumors with deleterious mutations in breast cancer gene 1 (*BRCA1*) or 2 (*BRCA2*) or other alterations associated with homologous recombination deficiency, inhibition of PARP results in cell death via synthetic lethality [2, 3]. In the United States, rucaparib is approved as a monotherapy treatment for patients with *BRCA* mutation (germline and/or somatic)–associated advanced ovarian cancer who have been treated with two or more chemotherapies [1]. In the European Union, rucaparib is approved as a monotherapy treatment for adult patients with platinum-sensitive, relapsed or progressive, *BRCA*-mutated (germline and/or somatic) epithelial ovarian, fallopian tube, or primary peritoneal cancer who have been treated with two or more prior lines of platinum-based chemotherapy and who are unable to tolerate further platinum-based chemotherapy [4]. Rucaparib is also approved in both the United States and the European Union as a monotherapy maintenance treatment for adult patients with recurrent, high-grade epithelial ovarian, fallopian tube, or primary peritoneal cancer who are in response (complete or partial) to platinum-based chemotherapy [1, 4]. Moreover, rucaparib is being investigated for treatment of other solid tumors, including prostate cancer (NCT02952534 and NCT02975934).

The pharmacokinetics (PK) of rucaparib was evaluated in CO-338-010 (Study 10; NCT01482715), a phase I/II study in patients with advanced solid tumors [5, 6]. Plasma exposure was dose proportional across dose ranges of 40–500 mg once daily (QD) and 240–840 mg twice daily (BID); median time to maximum concentration (t_max_) ranged 1.5–6.0 h and 1.5–4.0 h after QD and BID dosing, respectively. Apparent steady-state clearance was comparable with QD (26.7–47.5 L/h) and BID dosing (26.2–58.6 L/h).

At the recommended phase II dose of 600 mg BID, steady state was achieved following 1 week of dosing, with approximately four-fold accumulation. A high-fat meal increased maximum concentration (C_max_) and area under the concentration-time curve (AUC) of rucaparib by 20% and 38%, respectively, following a single oral dose of rucaparib 600 mg as compared with those under fasted conditions. The food effect was not considered clinically significant.

Preclinical studies investigating the metabolism of rucaparib indicate that rucaparib is metabolized by cytochrome P450 (CYP) 2D6, CYP1A2, and CYP3A4. The carboxylic acid M324, an oxidative metabolite of rucaparib, was observed in cryopreserved hepatocytes from rats, dogs, monkeys, and humans [7]. Preliminary metabolite profiling in plasma samples collected from three patients with cancer who enrolled in Study 10 and were treated with rucaparib 600 mg BID suggested that M324 is a major metabolite of rucaparib [6]. In an effort to further characterize the clinical PK profile of rucaparib, we initiated a two-part absorption, distribution, metabolism, and excretion (ADME) study (Study CO-338-045 [NCT02986100]). We administered a single oral dose of [^14^C]-rucaparib to patients with solid tumors to identify the pathways that are involved with the metabolism of rucaparib and the major routes of excretion for rucaparib and its metabolites, including metabolite M324. Results from Study CO-338-045 Part 1 are reported herein.

## Materials and methods

### Study design

This was a phase I, open-label, ADME study in patients with histologically or cytologically confirmed advanced solid tumors. The study consisted of two parts: an ADME study in patients with solid tumors associated with a deleterious *BRCA* mutation (Part 1, reported here) and continuous rucaparib treatment (Part 2) in patients who completed Part 1.

Six patients received a single oral dose of [^14^C]-rucaparib 600 mg (approximately 5.18 MBq or 140 μCi) on day 1 after fasting for 10 h and were confined at the study site for the collection of blood samples and excreta for a maximum of 12 days.

The study was conducted in accordance with the International Conference on Harmonisation Good Clinical Practice guidelines and the Declaration of Helsinki. The protocol was approved by the Ethics Committee for Clinical Pharmacology of Medical Research Council in Hungary. All patients provided written informed consent. The study was registered at ClinicalTrials.gov as NCT02986100.

### Patients

All patients in Part 1 had to meet the following inclusion criteria to enter and participate in the study: (1) ≥18 years of age with a histologically or cytologically confirmed advanced solid tumor; (2) able to understand and willing to sign the informed consent form and to comply with the study restrictions; (3) an Eastern Cooperative Oncology Group Performance Status of 0 or 1 and a life expectancy of ≥3 months; and (4) adequate body mass index (18.0–35.0 kg/m^2^), bone marrow function (absolute neutrophil count ≥1500/μL, platelets ≥100,000/μL, and hemoglobin ≥9 g/dL), renal function (glomerular filtration rate ≥45 mL/min using the Cockcroft Gault formula), and hepatic function (bilirubin ≤1.5 × upper limit of normal [ULN] or ≤2 × ULN if hyperbilirubinemia is due to Gilbert’s syndrome, alanine transaminase and aspartate aminotransferase ≤3 × ULN, and serum albumin ≥3 g/dL).

Patients were excluded from the study if they: (1) had acute illness, blood loss (>450 mL), active second malignancy or infections, or had undergone cancer therapy, such as chemotherapy, within 2 weeks of rucaparib administration and/or had ongoing adverse effects from such treatment; (2) participated in a trial involving administration of [^14^C]-labeled compounds within the last 6 months prior to day 1, participated in another investigational drug trial within 14 days prior to day 1, or had exposure to more than 3 new investigational agents within 12 months prior to day 1; (3) had a nonstudy-related minor surgical procedure ≤5 days or major surgical procedure ≤21 days prior to day 1; (4) had preexisting gastrointestinal disorders that would interfere with absorption of rucaparib, irregular bowel habits, any prior exposure to rucaparib, or a history of allergy, hypersensitivity, or idiosyncratic reaction to rucaparib or to any excipients present in the drug product; (5) had known hepatitis B virus, hepatitis C, or HIV infection; (6) had untreated or symptomatic central nervous system metastases, clinically significant arrhythmias, clinically significant electrocardiogram (ECG) abnormalities, QTcF interval ≥480 msec, arterial or venous thrombi, myocardial infarction, hospital admission for unstable angina, or cardiac angioplasty; or (7) were pregnant or breastfeeding.

Patients were prohibited from taking certain medications or treatments (e.g., chemotherapy, radiation, antibody therapy or other immunotherapy, gene therapy, vaccine therapy, angiogenesis inhibitors, alcohol, or antibiotics) or any herbal supplement or fruits (e.g., grapefruit) that might affect the ADME of rucaparib. Adequate use of birth control was mandatory for the duration of the study.

### Study medication and dosage

The investigational medicinal product was manufactured in the designated pharmacy (Pharmacy of PRA Health Sciences, Groningen, The Netherlands) by mixing nonradiolabeled and radiolabeled rucaparib camsylate salt (Supplemental Fig. 1) into hard gelatin capsules (≈150 mg rucaparib [freebase weight]; ≈1.295 MBq [35 μCi] of [^14^C]-rucaparib). The [^14^C]-rucaparib was characterized by Pharmaron (Rushden. Northamptonshire, UK), and the unlabeled rucaparib was characterized by Lonza (Basel, Switzerland). The chemical purities of both radiolabeled and nonradiolabeled rucaparib were >98%.

### Safety assessment

The safety of rucaparib was evaluated during the study. The National Cancer Institute Common Terminology Criteria for Adverse Events (CTCAE) grading system (version 4.03) was used to describe the severity of the adverse events (AEs) that occurred. Clinical laboratory, vital sign, 12-lead ECG, and physical examinations were also part of the safety assessment.

### Chemicals

Trifluoroacetic acid (TFA), dimethylsulfoxide (DMSO), and acetonitrile used for chromatographic analysis were high-performance liquid chromatography (HPLC) grade or above and were purchased from Thermo Fisher (Waltham, MA, USA) or another commercial supplier. Combustaid, Carbo-Sorb E absorber, PermaFluor E, Ultima Gold, and Ultima-Flo M scintillation fluids were obtained from PerkinElmer (Waltham, MA, USA). All other reagents were analytical or American Chemical Society reagent grade.

### Sample collection

Blood samples were collected in K_2_ ethylenediaminetetraacetic acid (K_2_-EDTA) tubes at the following time points: 0 (predose), 1, 2, 3, 4, 8, 12, 24, 48, 72, 96, 120, and 144 h after administration. Blood samples were taken via an indwelling intravenous catheter or by direct vein puncture into appropriate tubes. Aliquots of blood samples were centrifuged at 1600–2000×g for 10 min at 4 °C, and supernatants were collected for plasma samples. All plasma and blood samples were stored at −70 °C until analysis. Plasma and blood samples were evaluated for total radioactivity (TRA) by liquid scintillation (LSC), parent drug concentrations by liquid chromatography (LC) with tandem mass spectrometry (MS/MS) analyses, and metabolite profiling by LC coupled to diode array ultraviolet (UV) detection and MS or radioactivity detection.

For the purposes of the mass balance study and metabolite profiling, urine and fecal samples were collected during predetermined intervals. Urine was collected at time 0 (predose), 0–6, 6–12, and 12–24 h, and daily per 24-h interval from day 2 to day 12. Urine was collected in 3-L polyethylene containers and stored at 4 °C for the duration of the collection period. After each collection period, urine samples were mixed thoroughly, weighed, portioned into aliquots in polypropylene tubes, and then frozen at or below −70 °C until metabolite profiling and TRA analyses by LSC. Feces were collected daily in 24-h intervals from predose to day 12. Feces collections were kept at or below −20 °C. Sample pretreatment involved diluting and homogenizing the feces by adding water (1–2 weight equivalents). An Ultra-Turrax mixer (IKA, Staufen im Breisgau, Germany) was used to homogenize the samples before aliquoting. Separate aliquots of fecal homogenate were transferred to polypropylene tubes. Aliquots for radioactivity measurement by LSC were stored at or below −20 °C until analysis, whereas aliquots for metabolite profiling were stored at −70 °C until analysis.

Patients 01 and 05 vomited within the first 24 h post dose. The vomit was collected, weighed, and homogenized using an Ultra-Turrax mixer. Separate aliquots were analyzed for TRA by LSC.

Patients 01, 05, and 09 were confined to the study site for sample collections for 12 days, but Patients 03, 06, and 08 were discharged earlier per protocol because cumulative recovery of radioactivity from these patients exceeded 90% of the administered dose or the radioactivity in urine and feces was <1% of the administered dose over a 24-h period on 2 consecutive days.

### Total radioactivity analysis

TRA analysis was performed on a Liquid Scintillation Counter Tri-Carb Model 3100TR (PerkinElmer). Aliquots of blood (300 μL), plasma (250 μL), and urine (1 mL) were transferred to scintillation vials. Blood samples were incubated at ≥60 °C with SOLVABLE (1 mL). After cooling to room temperature, EDTA (0.1 M, 100 μL) and hydrogen peroxide (225 μL) were added to dissolve blood cells, reduce foaming, and decrease color intensity. Finally, after incubation at ≥45 °C and ≥60 °C, Ultima Gold (≥5-fold) was added, and the samples were analyzed with LSC. For urine samples, Ultima Gold (≥5-fold) was added, and the samples were analyzed with LSC.

Fecal samples were processed further and combusted before LSC analyses. Aliquots (≈500 mg) of the fecal homogenates were dried in an oven (50 °C). Combustaid (100 μL) was added to the dry homogenates and the sample was combusted in a Sample Oxidizer Model 307 (PerkinElmer). Carbo-Sorb E (7 mL) was used as an absorber agent for the [^14^C]-carbon dioxide that was generated during combustion. At the end of the combustion cycle, the absorber was mixed with 13 mL of the scintillant PermaFluor E. After combustion, TRA of fecal aliquots was determined by LSC as described for urine samples.

For the vomit sample collected from Patient 01 (clear vomitus) the urine method was used, and for Patient 05 (not clear vomitus) the feces method was used.

### Quantification of rucaparib in plasma

Plasma concentrations of rucaparib were determined by Q^2^ Solutions (Ithaca, NY, USA) using a validated LC-MS/MS method as previously described [6]. The concentration range for quantitation was 5–10,000 ng/mL.

### Pharmacokinetic analysis

Blood and plasma TRA data and the plasma concentration of rucaparib were used to determine the C_max_, t_max_, AUC from time zero to time of last measurable concentration (AUC_0-t_), and AUC from time zero to infinity (AUC_0-inf_), apparent volume of distribution (Vd/F), apparent clearance (CL/F), and terminal half-life (t_1/2_). Phoenix WinNonlin software version 6.3 or higher (Certara, Princeton, NJ, USA) was used to run a noncompartmental method analysis on the PK data.

### Metabolite profiling in plasma, urine, and feces

#### Preparation of plasma

Plasma samples from six patients were pooled using two methods: (1) A Hamilton pool plasma (0–24 h) for metabolite profile determination was prepared by combining plasma aliquots of a volume proportional to the time interval used for calculating the AUC (AUC_0-24h_ pool) for each patient [8] and (2) four time-point pooling samples were obtained by equal volume pool at 1, 4, 8, and 24 h across six patients. In addition, plasma samples at 1, 8, and 24 h were selected from three patients (Patients 03, 08, and 09) for metabolite profiles analysis to check the individual differences between patients. Samples collected after 24 h post dose were not included in the pooling due to the low radioactivity at later time points. All plasma samples (pooled and from individuals) were extracted at least two times with acetonitrile. All supernatants were evaporated to dryness, and the dried residues were reconstituted in 20% acetonitrile in water containing 0.1% TFA. The sample recovery was obtained by comparing the radioactivity concentrations before and after extraction, and the mean recovery was about 70%.

Aliquots of the supernatants were injected into the HPLC, and the HPLC elutes were collected as fractions at intervals of 30 sec per well into LumaPlate-96 DeepWell microplates (PerkinElmer). Due to the low radioactivity in each plasma sample, metabolite profiles were obtained by TopCount (PerkinElmer) analysis of HPLC elutes. The fractions were dried at room temperature, and then analyzed by a TopCount NXT radiometric microplate reader (PerkinElmer) to obtain metabolite profiles. The supernatants of extracts were also analyzed using an LC-MS method to identify metabolites.

#### Preparation of urine

For each patient, urine samples from different collection intervals were pooled in proportion to their sample weight in order to obtain a pooled sample covering 90% of the TRA in urine. Pooled urine samples were lyophilized and then extracted at least twice with acetonitrile. The obtained supernatant was evaporated to dryness, and the dried residues were reconstituted in 20% acetonitrile in water containing 0.1% TFA. An aliquot of the supernatant was analyzed for TRA to determine extraction efficiency, and the mean recovery was ≈70%. Aliquots of the supernatants were injected into the HPLC coupled to UV and radioactive detectors to obtain metabolite profiles. The samples were also analyzed using LC-MS for metabolite identification.

#### Preparation of feces

For each patient, homogenized feces samples from different collection intervals were pooled in proportion to their sample weight in order to obtain a pooled sample covering 90% of the TRA recovered in feces. Aliquots of the pooled fecal samples were extracted at least twice with acetonitrile. The obtained supernatant was evaporated. The dried residues were reconstituted in 40% DMSO in water to obtain maximum recovery. An aliquot of the supernatant was analyzed for TRA to determine extraction efficiency, and the mean recovery was ≈75%. Aliquots of the supernatants were injected into the HPLC coupled to UV and radioactive detectors to obtain metabolite profiles. The sample supernatants were also analyzed using LC-MS to identify metabolites.

### LC-MS-radioactivity systems for metabolite profiling and identification

Metabolite profiling and identification were performed on an LC system (Agilent) coupled to an LTQ Orbitrap mass spectrometer (Thermo Electron, Waltham, MA, USA) in combination with offline TopCount (LC-MS-TopCount) or online Radiomatic 625TR (LC-MS-Radiomatic; PerkinElmer). Proposed structures of the metabolites were based on accurate mass (<5 ppm) and comparison of mass spectral data of parent compound rucaparib and metabolite standard M324. The Radiomatic 625TR flow detector, equipped with a 200 μL flow cell, was operated using scintillation cocktail (Ultima-Flo M) delivered at a flow rate of 1 mL/min. Chromatography was done using a Phenomenex (Torrance, CA, USA) Luna Phenyl-Hexyl column (250 × 2 mm, 5 μm, 100 Å) at ambient temperature during the sample analysis. The mobile phase was (A) 0.05% TFA in water and (B) 0.05% TFA in acetonitrile, with a 0.2 mL/min flow rate. The gradient was as follows: 5% B over 2.1 min, to 20% B in 1.0 min, to 35% B in 16.9 min, isocratic at 35% B for 5 min, to 95% B over 5 min, and reduced to 5% B over 1 min, with total run time 45 min.

The LTQ Orbitrap mass spectrometer was equipped with an electrospray ionization interface and operated in positive ionization mode for metabolite profiling and identification. Mass spectra were acquired in full scan (MS) (*m/z* 150–1500) and data-dependent scan modes. Mass spectrometer parameter settings were: spray voltage, +5.0 kV; capillary temperature, 350 °C; sheath gas, 80 (arbitrary unit); auxiliary gas, 30 (arbitrary unit); activation Q, 0.25; activation time, 30 msec; resolution, 7500; and collision energy, 35 eV.

### Data analysis and calculations

Xcalibur (version 2.1; Thermo Fisher) was used to acquire mass spectral data on the LTQ Orbitrap LC-MS system. Flo-One (version 3.65; Packard Instrument Company, Meriden, CT, USA) was used to control the Radiomatic 625TR and acquire radiochromatograms. TopCount NXT was operated by Windows NT-based Hologram relational database software (Microsoft, Redmond, WA, USA). QuantaSmart (PerkinElmer) was used to acquire TRA for LSC. Analyst 1.6.2 was used for the LC-MS (API 4000) data process.

Statistical analyses were limited to simple calculations of variation, including mean and standard deviation (SD), as appropriate. The quantitative data were generated using Microsoft Excel and rounded to three significant figures. PK parameters (including C_max_, t_max_, t_1/2_, AUC, CL/F, and Vd/F) were calculated using WinNonlin software (version 6.2 or higher).

### Identification and characterization of metabolites

The structures of the metabolites were identified by LC-MS/MS based on comparisons of mass spectral fragmentation patterns with those produced by the parent compound. Further confirmation of the identities of the metabolites was based on the elemental composition determined from accurate mass analyses using an Orbitrap high-resolution mass spectrometer.

The structures of the major metabolites found in plasma, urine, and feces were also confirmed by comparisons with the synthetic reference standard M324.

### Rucaparib and metabolite abundance estimation

The abundance of unchanged rucaparib and metabolites identified in plasma, urine, and feces was determined as a percentage of the TRA in the analyzed samples. Percentage of the administered dose of rucaparib and metabolites was calculated in urine and feces (formula A). The concentration of rucaparib and its metabolites in plasma (ng Eq h/mL) was calculated based on the specific activity, TRA, and percentage of peaks (formula B). The AUC_0-24h_ value of rucaparib and its major metabolite M324 in plasma was calculated using both Hamilton method (formula C) and linear trapezoidal method (formula D).

(A) $$ \%\mathrm{Dose}\ \mathrm{of}\ \mathrm{metabolite}=\mathrm{TRA}\ \mathrm{in}\ \mathrm{urine}\ \mathrm{or}\ \mathrm{feces}\ \left(\mathrm{dpm}\right)\div \mathrm{TRA}\ \mathrm{in}\ \mathrm{administered}\ \mathrm{dose}\left(\mathrm{dpm}\right)\times \%\mathrm{metabolites}\ \mathrm{in}\ \mathrm{matrix} $$
(B) $$ \mathrm{Metabolite}\ \mathrm{concentrations}\ \mathrm{in}\ \mathrm{plasma}\ \left(\mathrm{ng}\ \mathrm{Eq}/\mathrm{mL}\right)=\mathrm{TRA}\ \mathrm{in}\ \mathrm{plasma}\ \left(\mathrm{dpm}/\mathrm{mL}\right)\ \mathrm{specific}\ \mathrm{activity}\left(\mathrm{ng}\ \mathrm{Eq}/\mathrm{dpm}\right)\times \%\mathrm{metabolites}\ \mathrm{in}\ \mathrm{the}\ \mathrm{matrix} $$
(C) $$ \mathrm{Hamilton}\ \mathrm{method}:{\mathrm{AUC}}_{0-24\mathrm{h}}\ \left(\mathrm{ng}\ \mathrm{Eq}\ \mathrm{h}/\mathrm{mL}\right)=\mathrm{concentration}\ \mathrm{in}\ \mathrm{Hamilton}\ \left({\mathrm{AUC}}_{0-24\mathrm{h}}\right)\mathrm{pooled}\ \mathrm{plasma}\ \left(\mathrm{ng}\ \mathrm{Eq}/\mathrm{mL}\right)\times 24\left(\mathrm{h}\right) $$
(D) $$ \mathrm{Linear}\ \mathrm{trapezoidal}\ \mathrm{method}:{\mathrm{AUC}}_{0-24\mathrm{h}}\left(\mathrm{ng}\ \mathrm{Eq}\ \mathrm{h}/\mathrm{mL}\right)=\frac{{\mathrm{C}}_{1\mathrm{h}}}{2}\times 1\mathrm{h}+\frac{{\mathrm{C}}_{1\mathrm{h}}+{\mathrm{C}}_{4\mathrm{h}}}{2}\times \left(4\mathrm{h}-1\mathrm{h}\right)+\frac{{\mathrm{C}}_{4\mathrm{h}}+{\mathrm{C}}_{8\mathrm{h}}}{2}\times \left(8\mathrm{h}-4\mathrm{h}\right)+\frac{{\mathrm{C}}_{8\mathrm{h}}+{\mathrm{C}}_{24\mathrm{h}}}{2}\times \left(24\mathrm{h}-8\mathrm{h}\right) $$

## Results

### Patients

Six female patients (white and not of Hispanic or Latino ethnicity), five with breast cancer and one with vaginal cancer, were enrolled in the ADME portion of the study (Part 1). Baseline characteristics are shown in Table 1. All patients carried the *BRCA* mutation (*BRCA1*, *N* = 4; *BRCA2*, *N* = 2). All six patients completed Part 1 of the study.

**Table 1:** Baseline characteristics

Characteristic	Patients (*N* = 6)
Age, years	
Median (range)	61 (38–67)
Sex, *n* (%)	
Female	6 (100)
Race, *n* (%)	
White	6 (100)
Weight, kg	
Mean (SD)	62.2 (11.0)
Height, cm	
Mean (SD)	165.3 (6.3)
BMI, kg/m^2^	
Mean (SD)	22.7 (3.3)
ECOG PS, *n* (%)	
0	1 (16.7)
1	5 (83.3)
Smoking status, *n* (%)	
Current	2 (33.3)
Nonsmoker	4 (66.7)

*BMI* body-mass index, *ECOG PS* Eastern Cooperative Oncology Group Performance Status, *SD* standard deviation

### Safety

There were a total of five treatment-emergent AEs (TEAEs) in four patients. The TEAEs included two events of vomiting in two patients (33.3%). No TEAE was considered to be related to the study drug by the investigators, had a severity of more than CTCAE grade 2, led to a withdrawal of the patient, or was a serious AE.

Laboratory parameters, vital signs, 12-lead ECG recordings, and physical examinations did not indicate remarkable trends or deviations. Individual abnormalities of the assessed safety parameters were generally not clinically significant.

### Mass balance

**Fig. 1:**
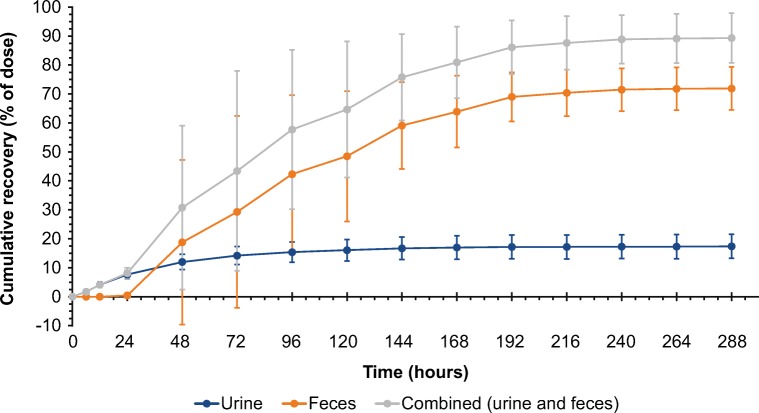
Cumulative excretion in excreta after a single oral dose of [^**14**^C]-rucaparib 600 mg administered to patients with solid tumors. Vertical bars represent the standard deviation of the arithmetic mean

As shown in Fig. 1, total dosed [^14^C]-rucaparib was almost completely excreted out of the human body within 12 days post dose, with a mean ± SD total of 89.3 ± 8.54% of the administered radioactive dose recovered in excreta (71.9 ± 7.40% in feces; 17.4 ± 4.17% in urine). Ninety percent of the observed urinary recovery was achieved by 120 h post dose, and 90% of the observed fecal recovery was found within 168 h post dose. For Patients 01 and 05, who vomited during the first 24 h post dose, the amount of radioactivity of the vomit samples was determined and used to calculate the actual dose (i.e., dose administered minus the loss due to vomiting) and fraction excreted into urine and feces. Vomiting led to a loss of 31.6% and 0.03% of the radioactivity of the administered dose in Patients 01 and 05, respectively. Patient 01 had 86.5% cumulative recovery of the actual dose, which was comparable with the other patients. Thus, this patient was considered evaluable.

### Pharmacokinetics

**Fig. 2:**
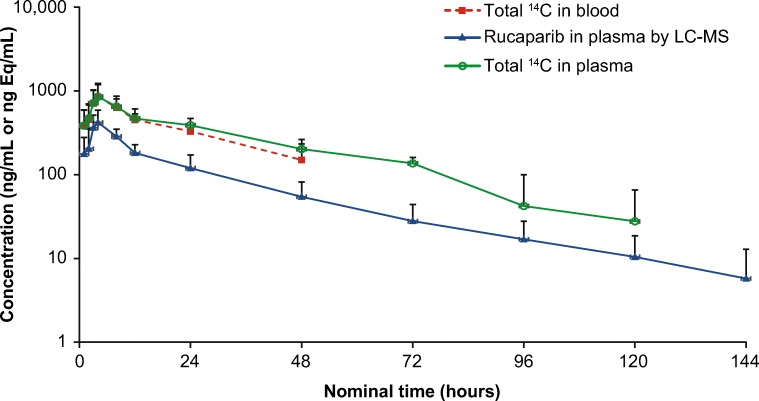
Plasma and blood after a single oral dose of [^**14**^C]-rucaparib 600 mg administered to patients with solid tumors. Concentration data are presented as arithmetic mean (standard deviation). Abbreviation: *LC-MS* liquid chromatography mass spectrometry

The concentration-time profiles of TRA in plasma and blood, along with unchanged rucaparib in plasma are shown in Fig. 2. The plasma TRA-time profile was parallel with but higher than that of rucaparib, suggesting that rucaparib metabolites were formed. A summary of PK parameters of TRA in plasma and blood and unchanged rucaparib in plasma is presented in Table 2. The mean C_max_ of TRA in plasma and blood and unchanged rucaparib in plasma was reached at a median t_max_ of 4 h post dose. Thereafter, the concentrations declined slowly, with elimination t_1/2_ > 25 h. The blood-to-plasma ratios of TRA were 1.00 for C_max_ and 0.781 for AUC_0-inf_, suggesting that rucaparib and its related metabolites had limited penetration in red blood cells. The mean CL/F and the Vd/F of rucaparib in plasma were higher than those of TRA in plasma.

**Table 2:** Summary of pharmacokinetic parameters of total radioactivity and unchanged rucaparib

Parameter	Total radioactivity in blood and plasma, mean ± SD			Rucaparib concentration in plasma, mean ± SD
	Plasma	Blood	Blood to plasma ratio	
C_max_, ng Eq/mL or ng/mL	880 ± 309	882 ± 355	1.00 ± 0.12	428 ± 154
t_max_, h^a^	4 (0.933–8.00)	4 (0.933–8.00)	–	4 (0.933–8.00)
AUC_0-t_, ng Eq h/mL or ng h/mL	24,100 ± 8580	18,100 ± 5460	–	8950 ± 3230
AUC_0-inf_, ng Eq h/mL or ng h/mL	30,000 ± 7810	25,500 ± 5510	0.781 ± 0.041	9340 ± 3480
t_1/2_, h	30.4 ± 8.89	28.3 ± 8.49	–	25.9 ± 10.1
CL/F, L/h	19.6 ± 4.83	22.6 ± 2.22	–	73.9 ± 45.5
Vd/F, L	814 ± 129	911 ± 246	–	2300 ± 490

^a^t_max_ presented as median (range)

*AUC*_*0-inf*_ area under the concentration-time curve from time zero to infinity, *AUC*_*0-t*_ area under the concentration time curve from time zero to time of last measurable concentration, *CL/F* apparent clearance, *C*_*max*_ maximum concentration, *SD* standard deviation, *t*_*1/2*_ terminal half-life, *t*_*max*_ time to maximum concentration, *Vd/F* apparent volume of distribution

### Metabolite profiles

**Fig. 3:**
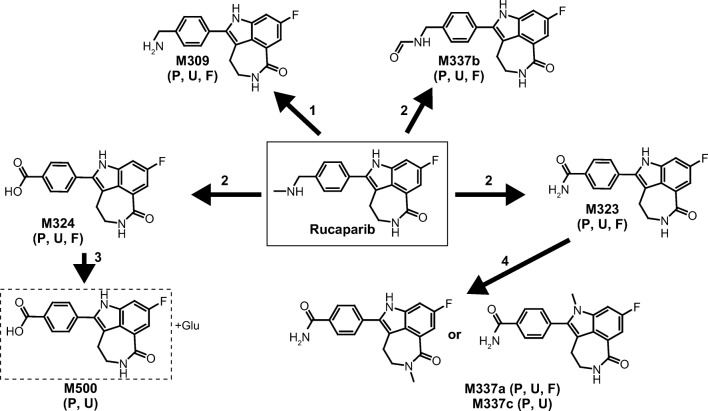
Proposed metabolic pathways of rucaparib following a single oral dose of [^**14**^C]-rucaparib 600 mg. Numbered arrows denote the following: 1, *N*-demethylation; 2, oxidation; 3, glucuronidation; and 4, *N*-methylation. The dashed-line box indicates that the position of the glucuronide on M324 cannot be concluded. Abbreviations: *F* feces; *P* plasma; *U* urine

Metabolite profiling was conducted in plasma, urine, and feces to determine the metabolism of rucaparib in patients with cancer following a single oral dose of [^14^C]-rucaparib 600 mg. Rucaparib was found to be metabolized via oxidation, *N*-demethylation, *N*-methylation, and glucuronidation in humans (Fig. 3). Seven metabolites were identified in plasma, urine, and feces (Table 3): M309, M323, M324, M337a, M337b, M337c, and M500. Unchanged rucaparib and M324 were the major drug-related components in all matrices. The peak distribution and abundance of rucaparib and metabolites in pooled plasma are listed in Table 4 and those in urine and feces are shown in Supplemental Table 1. Representative plasma, urine, and feces LC radiochromatograms and LC-MS chromatograms are presented in Supplemental Figs. 2–5.

**Table 3:** Summary of rucaparib metabolites identified in plasma, urine, and feces of patients administered a single oral dose of [^**14**^C]-rucaparib 600 mg

Compound name	Retention time, min	Biotransformation	Formula (MH^+^)	Accurate mass (MH^+^) *m/z*			Matrix	Fragment ions
				Experimental	Theoretical	*ppm*		
Rucaparib	15	Parent	C_19_H_19_FN_3_O	324.1500	324.1507	−2.2	P, U, F	293, 276, 264, 237
M324	28	Oxidation	C_18_H_14_FN_2_O_3_	325.0980	325.0983	−0.9	P, U, F	282, 238
M309	14	*N*-demethylation	C_18_H_17_FN_3_O	310.1347	310.1350	−1.0	P, U, F	293, 276, 237
M500	19–20	Oxidation, glucuronidation	C_24_H_22_FN_2_O_10_	501.1299	501.1304	−1.0	P, U	325, 282, 238
M323	21	Oxidation	C_18_H_15_FN_3_O_2_	324.1137	324.1143	−1.9	P, U, F	307, 281, 264, 238
M337c	22	Oxidation, *N*-methylation	C_19_H_17_FN_3_O_2_	338.1307	338.1299	2.4	P, U	321, 292, 264
M337a	23	Oxidation, *N*-methylation	C_19_H_17_FN_3_O_2_	338.1301	338.1299	0.6	P, U, F	295, 238, 264
M337b	24	Oxidation	C_19_H_17_FN_3_O_2_	338.1296	338.1299	−0.9	P, U, F	293, 237

*F* feces, *P* plasma, *U* urine

**Table 4:** Summary of peak distribution and abundancy of rucaparib and metabolites in pooled plasma up to 24 h

Radioactive peaks^a^	Hamilton (AUC_0-24h_) pooled plasma (*N* = 6)			Time point pooled plasma^b^				
	% Peak, mean ± SD	Concentration, ng Eq/mL	AUC_0-24h_,^c^ ng Eq h/mL	Mean concentration, ng Eq/mL				Mean AUC_0-24h_,^c^ ng Eq h/mL
				1 h	4 h	8 h	24 h	
Rucaparib	64.0 ± 13.7	335 ± 120	8040	317	590	545	151	9360
M324	18.6 ± 10.8	94.0 ± 62.7	2260	31.1	265	105	239	3960

^a^Other metabolites M309, M323, M337a, M337b, M337c, and M500 were detected in small to trace amounts and were not included

^b^For each time point, a single sample of pooled plasma from all six patients was analyzed

^c^AUC_0-24h_ in Hamilton pool = concentration in 0–24-h pooled sample (ng Eq/mL) × 24 h, and AUC_0-24h_ from the time point pool was calculated with WinNonlin software using the trapezoidal method

*AUC*_*0-24h*_ area under the concentration-time curve from 0 to 24 h, *SD* standard deviation

#### Metabolite profiles in plasma

In the Hamilton AUC_0-24h_ pooled plasma, unchanged rucaparib and M324 were the major drug-related components identified. The mean ± SD of peak distribution of rucaparib and M324 was 64.0 ± 13.7% and 18.6 ± 10.8% of plasma radioactivity, respectively. M309, M323, M337a, M337b, M337c, and M500 were detected in small or trace amounts, and together accounted for 13.8 ± 16.9% of TRA in plasma. Qualitatively similar metabolite profiles were obtained in the time point pooled samples from all six patients at 1, 4, 8, and 24 h (Table 4), as were individual plasma samples from the three selected patients (Patients 03, 08, and 09) at 1, 8, and 24 h (Supplemental Table 2); unchanged rucaparib and M324 were the major drug-related components in all plasma examined in this study (data not shown). Based on the peak distributions, TRA in plasma, and specific activity of the dose formulation of [^14^C]-rucaparib, concentrations of major radioactive peaks were calculated and expressed in ng Eq/mL. The mean ± SD concentrations and calculated AUC_0-24h_ values were 335 ± 120 ng Eq/mL and 8040 ng Eq h/mL for rucaparib and 94.0 ± 62.7 ng Eq/mL and 2260 ng Eq h/mL for M324, respectively (Table 4). The major circulated metabolite M324 was detected as 30.3% of unchanged rucaparib exposure in 24-h pooled plasma. Using the same method, concentrations of rucaparib and M324 at the 1-, 4-, 8-, and 24-h time points were calculated (Table 4). The concentration of rucaparib reached its maximum at 4 h and gradually decreased over time, whereas M324 was formed over time. Using the four estimated concentrations at the 1-, 4-, 8-, and 24-h time points, AUC_0-24h_ values of rucaparib and M324 were calculated as 9360 and 3960 ng Eq h/mL, respectively, using the trapezoidal method (Table 4); this was comparable to the estimated AUC_0-24h_ values based on the Hamilton pooling method. To check the individual differences, the radioactive metabolite profiles of the plasma samples from the three selected patients were obtained at the 1-, 8-, and 24-h time points (Supplemental Table 2). There were no qualitative differences detected among the three selected patients; the time courses of mean percentages of rucaparib and M324 show that rucaparib decreased and M324 increased over time, which was consistent with the time-point pooled samples from all six patients.

#### Metabolite profiles in urine

In the pooled urine samples that represented 90% of urine radioactivity excretion, rucaparib and M324 were the major drug-related components for all six patients (Supplemental Table 1). Based on the peak integration and TRA excreted in urine from each patient, the distributions of major radioactive peaks in urine were calculated and expressed in percentages of the administered dose (Table 5). Rucaparib and M324 accounted for 44.9 ± 15.6% and 50.0 ± 15.5% of the TRA in urine and 7.59 ± 4.16% and 7.58 ± 1.61% of the administered dose, respectively. M309, M323, M337a, M337b, M337c, and M500 were observed in small to trace amounts and together accounted for <1% of the administered dose (Supplemental Table 1).

**Table 5:** Summary of peak distribution and abundancy of rucaparib and metabolites in excreta

Radioactive peaks	Urine		Feces	
	% Peak, mean ± SD	% Dose, mean ± SD	% Peak, mean ± SD	% Dose, mean ± SD
Rucaparib	44.9 ± 15.6	7.59 ± 4.16	94.9 ± 4.95	63.9 ± 8.60
M324	50.0 ± 15.5	7.58 ± 1.61	5.07 ± 4.95	3.29 ± 3.25
Other^a^	4.25 ± 2.52	0.70 ± 0.42	Trace	NC
Total	>99%	15.9 ± 3.89	>99%	67.2 ± 7.38

^a^Other metabolites include M309, M323, M337a, M337b, M337c, and M500

*NC* not calculated, *SD* standard deviation

#### Metabolites profiles in feces

In the pooled fecal homogenates that represented >90% of fecal excretion, rucaparib was the predominate component and accounted for 94.9 ± 4.95% of TRA in feces and 63.9 ± 8.60% of the administered dose, whereas M324 observed in plasma and urine samples accounted for 5.07 ± 4.95% of TRA in feces and 3.29 ± 3.25% of the administered dose in feces using the same calculation method that was utilized in pooled urine (Table 5 and Supplemental Table 1). A few metabolites, including M309, M323, M337a, and M337b, were detected in trace amounts in feces and mainly observed by MS only.

#### Metabolite identification

The chemical structures of each rucaparib metabolite identified and the proposed metabolism scheme based on the metabolite profiles in plasma, urine, and feces are shown in Fig. 3. The fragmentation patterns for rucaparib and metabolites can be found in Table 3.

The chemical structures were elucidated using MS^2^ or MS^3^ fragmentation. The MS^2^ spectra of rucaparib showed a product ion of *m/z* 293, which corresponded to the loss of methylamine. Further loss of NH_3_ from *m/z* 293 gave the fragment ion at *m/z* 276 in the MS^3^ spectra. Fragment ions at *m/z* 264 and *m/z* 237 in the MS^3^ spectra indicated cleavages on the azepanone ring. For M324, the fragment ion at *m/z* 282 in the MS^2^ spectra was proposed to be due to the cleavage of azepanone ring. Further loss of carboxylic acid moiety from *m/z* 282 gave the fragment ion at *m/z* 238 in the MS^3^ spectra. The chemical structures of M309, M323, M337a, M337b, M337c, and M500 observed in plasma, urine, and/or feces were identified by LC-MS/MS method.

The structures of rucaparib and M324 were also confirmed by comparing the mass spectra and retention time in each matrix with that of the reference standard. It should be noted that the structures of these metabolites other than M324 are tentative.

## Discussion

This study, conducted at a single study center in six female patients with various solid tumors, investigated the ADME of [^14^C]-rucaparib after a single oral dose of 600 mg. The plasma TRA-time profile was parallel to, but higher than that of unchanged rucaparib. The blood TRA-time profile was parallel to that in plasma, but was truncated after 48 h post dose due to the assay sensitivity limitation: the lower limit of quantitation in blood and plasma samples was 96.5–98.5 ng Eq/mL and 57.9–59.1 ng Eq/mL, respectively. The blood-to-plasma ratio of the TRA was approximately 0.781 in terms of AUC_0-inf_, suggesting that limited rucaparib-related components penetrated blood cells. The mean CL/F and the Vd/F of rucaparib were approximately 3.8- and 2.8-fold higher than that of TRA, respectively (Table 2), indicating that rucaparib was distributed extensively into human tissues; however, the distribution of metabolites was confined to plasma.

Two patients experienced vomiting. For these patients, the amount of radioactivity in vomitus was considered when calculating the actual dose and fraction excreted into urine and feces. Although one patient lost 31.6% of the radioactivity of the administered dose, this was not expected to affect the estimation of t_1/2_, CL/F, or Vd/F. However, no dose normalization was done for the calculation of C_max_ or AUC in the noncompartmental analysis. As a result, the reported C_max_ and AUC values represent the observed PK exposures in patients that correspond to the actual dose levels. The other patient lost 0.03% of the radioactivity of the administered dose and had the lowest cumulative dose recovery of 73.1% (10.9% in urine and 62.2% in feces). There were no other clinical observations for this patient (e.g., gastric abnormalities, concomitant medicines, or clinical AEs) that could explain the low dose recovery; thus, the exact reason for the low dose recovery remains unclear.

Rucaparib was metabolized in humans to form one major oxidative metabolite (M324) and six minor metabolites (M309, M323, M337a, M337b, M337c, and M500). These seven metabolites were detected in human plasma and urine, including in both phase I and phase II reactions, such as oxidation, *N*-demethylation, glucuronidation, and *N*-methylation. Unchanged rucaparib and M324 were the major radioactive components in all tested matrices (≈64% and ≈19% of radioactivity in plasma, >7% of administered dose in urine, ≈64% and 3.3% of administered dose in feces for rucaparib and M324, respectively), whereas the minor metabolites were detected in trace amounts and/or were observed by MS only. The M324 observed in this clinical study was also found to be a major rucaparib-related metabolite in three patients with solid tumors who participated in another clinical study investigating the PK profile of rucaparib [6]. In nonclinical models, M324 was also observed in rats and dogs.

Rucaparib and its metabolites were slowly eliminated from the human body, with a t_1/2_ of 30.4 and 25.9 h for TRA and unchanged rucaparib, respectively. The calculated long t_1/2_ of rucaparib was consistent with that in a previous clinical trial, where patients received a single oral dose of rucaparib (dose range, 40–500 mg; t_1/2_, 11–29.8 h) [6]. In the 24-h pooled plasma samples from our study, the concentration and percentage of rucaparib decreased over time, whereas those of M324 increased over time. Those changes were further observed in plasma samples from the three selected patients (Patients 03, 08, and 09; Supplemental Table 2). The samples collected after the 24-h postdose time point were of low concentrations (Fig. 2) and were not included in the metabolite profiling pool to avoid over dilution. The AUC_0-24h_/AUC_0-inf_ ratios were calculated to be 0.537, 0.461, and 0.563, respectively, for plasma unchanged rucaparib, plasma TRA, and blood TRA. Further investigation of the PK profile of M324 is ongoing in two clinical trials and will be reported separately.

The current study suggested an oral bioavailability of ≥21% based on the assumption that the total absorbed dose of [^14^C] radioactivity is equal to [^14^C] radioactivity recovered in urine plus [^14^C] radioactivity recovered as metabolites in feces. This assumption could lead to an underestimation of bioavailability as it does not account for rucaparib excreted in feces as intact drug and, therefore, explains the difference in oral bioavailability compared with the previously reported bioavailability of 36% [9]. In the mass balance study, 89.3% of the administered dose of rucaparib was recovered in urine (17.4%) and feces (71.9%) within 12 days. The high percentage of unchanged rucaparib in feces could be due to hepatic excretion pathways and/or unabsorbed dose. Given a 36% absolute oral bioavailability of rucaparib [9], renal and hepatic clearance were likely both major elimination pathways for rucaparib.

Metabolite profiling assessment determined that the peak distribution of rucaparib accounted for 64.0% (0.64) of radioactivity in 0–24-h pooled plasma samples (Table 4). The AUC_0-24h_ ratio of unlabeled rucaparib to TRA in plasma was 0.412 (Fig. 2). The former percentage/ratio was based on the peak integration of radioactivity profiles. The radioactive peaks were determined by LC-radioactivity detector. However, the unlabeled rucaparib concentration and TRA in the latter ratio were quantitatively analyzed by LC-MS/MS and LSC, respectively. Those different bioanalytical methods, with their own systemic errors in measurement, might lead to two variable ratios (0.640 vs 0.412). Others have observed that the ratio calculated by a metabolite profiling method can be higher than ratios calculated using bioanalysis assays [10, 11]. Minor rucaparib metabolites existing in small to trace amounts might not have been quantitatively determined by metabolite profiling assessment; however, these metabolites may have contributed to the plasma radioactivity and, therefore, overestimated the peak ratio of rucaparib to the radioactivity in 0–24-h plasma samples, which further explains the different ratios (0.640 vs 0.412).

In summary, following a single oral dose of [^14^C]-rucaparib, the C_max_ of TRA in plasma and blood and unchanged rucaparib in plasma was detected at approximately 4 h post dose and followed by parallel declines. The overall recovery of dosed radioactivity excreta was 89.3% over 12 days post dose (71.9% in feces and 17.4% in urine). Unchanged rucaparib and oxidative metabolite M324 were observed as the major components in plasma and urine, whereas rucaparib was observed as the predominant component in feces. The metabolic pathways of rucaparib in humans included oxidation, *N*-demethylation, *N*-methylation, and glucuronidation. Taken together, the results of this study suggest that rucaparib is eliminated through multiple pathways, which include metabolism as well as renal and biliary excretion.

## Electronic supplementary material

ESM 1 (DOCX 209 kb)
