# Supplementary material for: Evaluation of absorption, distribution, metabolism, and excretion of [14C]-rucaparib, a poly(ADP-ribose) polymerase inhibitor, in patients with advanced solid tumors
Source: Invest New Drugs. 2019 Jun 27;38(3):765–75. doi: 10.1007/s10637-019-00815-2 (PMC7211193; doi:10.1007/s10637-019-00815-2)
Supplement: Supplementary file 1 — (DOCX 209 kb) [file 10637_2019_815_MOESM1_ESM.docx]

# ELECTRONIC SUPPLEMENTARY MATERIAL

**Evaluation of absorption, distribution, metabolism, and excretion of [^14^C]-rucaparib, a poly(ADP-ribose) polymerase inhibitor, in patients with advanced solid tumors**

Mingxiang Liao^1^, Simon Watkins^1^, Eileen Nash^1^, Jeff Isaacson^1^, Jeff Etter^1^, Jeri Beltman^1^, Rong Fan^2^, Li Shen^2^, Abdul Mutlib^2^, Vendel Kemeny^3^, Zsuzsanna Pápai^4^, Pascal van Tilburg^5^, Jim J. Xiao^1^

^1^Clovis Oncology, Inc., 500 Flatiron Pkwy, Suite 100, Boulder, CO 80301, USA

^2^Frontage Laboratories, Inc., 100 Pennsylvania Dr., Exton, PA 19341, USA

^3^PRA Health Sciences, Rottenbiller utca 13, H-1077 Budapest, Hungary

^4^State Health Center, Róbert Károly krt. 44, 1134 Budapest, Hungary

^5^PRA Health Sciences, Bioanalytical Laboratory NL, Amerikaweg 18, 9407 TK Assen, The Netherlands

**Corresponding author:**

Jim J. Xiao, jxiao@clovisoncology.com, Telephone: 510-901-1578

## Supplemental Fig. 1 Molecular structure of [^14^C]-rucaparib camsylate

## Supplemental Fig. 2 Representative LC/radiochromatogram (a) and LC-MS chromatogram (b) of [^14^C]-rucaparib and metabolites in Hamilton (AUC_0-24h_) pooled plasma

*Detected by mass spectrometry only.

Abbreviations: *AUC_0-24h_* area under the concentration-time curve from 0 to 24 h; *LC-MS* liquid chromatography mass spectrometry

## Supplemental Fig. 3 Representative LC/radiochromatogram (a) and LC-MS chromatogram (b) of [^14^C]-rucaparib and metabolites in pooled urine up to 120 h

*Detected by mass spectrometry only.

Abbreviation: *LC-MS* liquid chromatography mass spectrometry

## Supplemental Fig. 4 Representative LC/radiochromatogram (a) and LC-MS chromatogram (b) of [^14^C]-rucaparib and metabolites in pooled feces up to 144 h

*Detected by mass spectrometry only.

Abbreviation: *LC-MS* liquid chromatography mass spectrometry

## Supplemental Fig. 5 LC-MS extracted ion chromatograms (XIC) of synthetic standards rucaparib and M324

Abbreviation: *LC-MS* liquid chromatography mass spectrometry

## Supplemental Table 1 Percentage of rucaparib dose and metabolites in pooled urine and pooled fecal samples from six human patients after a single oral administration of [^14^C]-rucaparib

| Compound name | Patient 01 | | Patient 03 | Patient 05 | Patient 06 | Patient 08 | Patient 09 | Mean ± SD |
| --- | --- | --- | --- | --- | --- | --- | --- | --- |
|  | % of dose of [^14^C]-rucaparib and metabolites in pooled urine (% dose) | | | | | | | |
| Rucaparib | 14.3 | | 5.03 | 2.85 | 5.96 | 10.7 | 6.77 | 7.59 ± 4.16 |
| M324 | 6.17 | | 7.24 | 7.22 | 8.54 | 6 | 10.3 | 7.58 ± 1.61 |
| Others^a^ | 1.15 | | 1.03 | 0.13 | 1.01 | 0.33 | 0.53 | 0.70 ± 0.42 |
| Total in urine | 21.6 | | 13.3 | 10.2 | 15.5 | 17 | 17.6 | 15.9 ± 3.89 |
|  | % of dose of [^14^C]-rucaparib and M324 in pooled fecal homogenate (% dose) | | | | | | | |
| Rucaparib | 50.6 | 66.8 | | 55.8 | 70.1 | 68.2 | 71.8 | 63.9 ± 8.60 |
| M324 | 6.39 | 7.82 | | 3.33 | 2.22 | 0.00 | 0.00 | 3.29 ± 3.25 |
| Total in feces | 57.0 | 74.6 | | 59.1 | 72.3 | 68.2 | 71.8 | 67.2 ± 7.38 |
| Total in excreta | 78.6 | 87.9 | | 69.3 | 87.8 | 85.2 | 89.4 | 83.1 ± 7.74 |

^a^Other metabolites include M309, M323, M337a, M337b, M337c, and M500.

Abbreviation: *SD* standard deviation

## Supplemental Table 2 Peak distribution of total radioactivity of rucaparib and metabolite M324 in individual plasma from Patients 03, 08, and 09 at 1, 8, and 24 h after a single oral administration of [^14^C]-rucaparib

| Time, h | % Radioactive peaks of rucaparib and M324 in individual plasma | | | | | | | |
| --- | --- | --- | --- | --- | --- | --- | --- | --- |
|  | Patient 03 | | Patient 08 | | Patient 09 | | Mean ± SD | |
|  | Rucaparib | M324 | Rucaparib | M324 | Rucaparib | M324 | Rucaparib | M324 |
| 1 | 75.0 | 15.4 | 92.1 | 7.94 | 84.6 | 15.4 | 83.9 ± 8.57 | 12.9 ± 4.31 |
| 8 | 66.7 | 33.3 | 68.2 | 20 | 82.1 | 17.9 | 72.3 ± 8.49 | 23.7 ± 8.35 |
| 24 | 36.1 | 63.9 | 38.5 | 61.5 | 56.5 | 36.2 | 43.7 ± 11.1 | 53.9 ± 15.3 |

Abbreviation: *SD* standard deviation
